# Disruption of CPSF6 enhances cellular permissivity to HIV-1 infection through alternative polyadenylation

**DOI:** 10.21203/rs.3.rs-5099896/v1

**Published:** 2024-12-04

**Authors:** Judd Hultquist, Daphne Cornish, Kathryn Jackson-Jones, Ted Ling-Hu, Lacy Simons, William Cisneros, Edmund Kuffour, Francesca Agnes, Yujin Lee, Paul Bieniasz, Ramon Lorenzo-Redondo

**Affiliations:** Northwestern University; Northwestern University Feinberg School of Medicine; Northwestern University Feinberg School of Medicine; Northwestern University Feinberg School of Medicine; Northwestern University; Northwestern University Feinberg School of Medicine; The Rockefeller University; Northwestern University Feinberg School of Medicine; Northwestern University Feinberg School of Medicine; Rockefeller University; Northwestern University

## Abstract

Human immunodeficiency virus (HIV) relies upon a broad array of host factors in order to replicate and evade the host antiviral response^1^. Cleavage and polyadenylation specificity factor 6 (CPSF6) is one such host factor that is recruited by incoming HIV-1 cores to regulate trafficking^2^, nuclear import^3–5^, uncoating^6^, and integration site selection^4,6–11^. Despite these well-described roles, the impact of CPSF6 perturbation on HIV-1 infectivity varies considerably by cell type. Here, we report that *CPSF6* knock-out in primary CD4+ T cells leads to increased permissivity to HIV-1 infection due to broad transcriptional reprogramming. Knock-out of *CPSF6* results in widespread differential gene expression, including downregulation of genes involved in the innate immune response and enhanced expression of the HIV-1 co-receptors. Accordingly, these cells are less responsive to interferon and express lower levels of antiretroviral restriction factors, including TRIM5α. These transcriptional changes are linked to global shortening of mRNA 3’ untranslated regions (UTRs) through alternative polyadenylation (APA), which is triggered by disruption of the CPSF6-containing Cleavage Factor Im (CFIm) complex^12,13^. Furthermore, we find that recruitment of CPSF6 by HIV-1 cores is sufficient to perturb CPSF6 function, leading to 3’ UTR shortening and subsequent transcriptional rewiring. These results suggest a novel mechanism by which HIV-1 transcriptionally reprograms CD4+ T cells through recruitment of CPSF6 to circumvent the innate immune response and enhance permissivity to infection.

## INTRODUCTION

The HIV-1 core is a metastable structure of capsid hexamers and pentamers that form a fullerene cone encapsulating two copies of the viral RNA genome^14^. After deposition into the host cell cytoplasm, the core shields the viral genome from innate immune sensing while facilitating reverse transcription and trafficking into the nucleus, whereupon it must disassemble to enable integration in a process known as uncoating. These functions are facilitated and counteracted by a number of host factors that bind to the core and regulate its form and function, including cyclophilin A (CYPA)^15–17^, tripartite motif-containing protein 5a (TRIM5α)^18,19^, nucleoporin 153 (NUP153)^20^, and cleavage and polyadenylation specificity factor 6 (CPSF6).

CPSF6 binds to incoming cores in the target cell and has been shown to regulate core trafficking to the nucleus^2^, nuclear import^3–5^, uncoating^21^, and integration site selection^4,6–11^. Additionally, CPSF6 has been proposed to promote immune evasion by shielding viral pathogen associated molecular patterns (PAMPs) from detection^22^. Despite these many roles in the viral lifecycle, reported HIV-1 infection phenotypes upon CPSF6 depletion vary widely by viral strain and cell type, including some conditions that result in an increase in infection^11,23^. Likewise, HIV-1 capsid mutants that can no longer bind CPSF6 (such as A77V and N74D) often yield conflicting and/or cell-type dependent phenotypes that may suggest pleiotropic effects^24–26^.

CPSF6 is a member of the cleavage factor Im (CFIm) complex alongside its binding partner, CPSF5^27^. The CFIm complex is a major regulator of polyadenylation site selection during host messenger RNA (mRNA) processing^28–30^. Disruption of CFIm function results in changes in a mechanism of post-transcriptional mRNA regulation known as alternative polyadenylation (APA), leading to a preference for proximal poly(A) sites and a global shortening of 3’ untranslated regions (UTRs)^12,13,31,32^. APA induction has several functional consequences within a cell, including changes in transcript abundance and localization^13^, and has been previously linked to regulation of the antiviral innate immune response^33^. It is unknown if APA influences the innate immune response to HIV-1 infection and if this phenomenon is related to the cell-type specific phenotypes observed upon CPSF6 depletion.

To better understand the role of CPSF6 in HIV-1 infection, we used CRISPR-Cas9 gene editing to knock-out *CPSF6* in primary CD4 + T cells. These cells were markedly more permissive to HIV-1 infection and had a dampened antiviral response. *CPSF6* knock-out resulted in global changes in APA, which correlated with decreased gene expression in innate immune pathways and increased expression of HIV-1 coreceptors. These cells were less responsive to type I interferon and had decreased expression of known antiretroviral restriction factors. Furthermore, HIV-1 infection was sufficient to perturb CPSF6 function and induce changes in APA. These studies suggest a model in which normal CPSF6 function is disrupted by its recruitment to incoming HIV-1 cores, allowing the virus to evade host antiviral responses by transcriptional reprogramming in a novel form of host shutoff.

## RESULTS

### *CPSF6* knock-out primary CD4+ T cells are more permissive to HIV-1 infection

**Figure 1:**
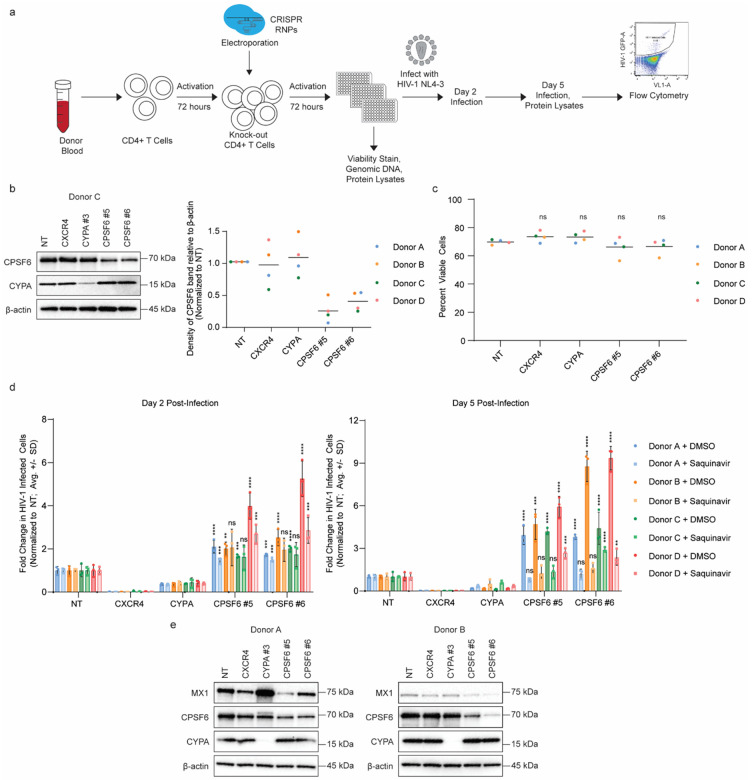
*CPSF6* knock-out in primary CD4+ T cells leads to increased HIV-1 infection rates and dampened innate immune induction. **a**, Experimental workflow for CRISPR-Cas9 editing of primary CD4+ T cells and downstream assays. CD4+ T cells were isolated from human blood, activated, and edited via electroporation of crRNPs. Edited cells were activated, expanded, and split into replica plates for knockout validation, viability stain, and spreading infection assays. For spreading infection assays, cells were infected with HIV-1 NL4–3 nef:IRES:GFP in technical triplicate per condition and harvested for analysis of infection rates by flow cytometry at days 2 and 5 post-infection. **b**, Immunoblot shows knock-out of *CPSF6* and *CYPA* in primary CD4+ T cell protein lysates harvested at day 4 post-editing in one representative biological replicate (donor C). Chart shows CPSF6 band density divided by β-actin band density in blots from Fig. 1b and Extended Data Fig. 1a in 4 biological replicates (donors A-D). Density measurements are normalized to non-targeting controls (NT) per donor, horizontal line shows average of biological replicates. **c**, Knock-out primary CD4+ T cells exhibit similar viability to NT controls at day four post-editing as assessed via amine dye stain and flow cytometry. Dots represent cell viability (% Ghost Red negative cells) per condition, horizontal lines represent the average of viability measurements in 4 biological replicates (donors A-D). Statistics were calculated relative to the NT control by two-way ANOVA with Dunnet’s test for multiple comparisons; ns = not significant. **d**, HIV-1 infectivity (% GFP positive cells) at days 2 (left) and 5 (right) post-challenge with HIV-1 NL4–3 nef:IRES:GFP in indicated knock-out primary CD4+ T cells from 4 biological replicates (donors A-D) as assessed by flow cytometry. Cells were treated with protease inhibitor (saquinavir) or DMSO control at 24 hours post-challenge as indicated. Each bar represents the average of technical triplicates +/− SD with individual data points shown. Statistics were calculated relative to the NT control per condition by one-way ANOVA with Dunnet’s test for multiple comparisons; * = p ≤ 0.05, ** = p ≤ 0.01, *** = p ≤ 0.001, **** = p ≤ 0.0001. **e**,Immunoblot showing expression of representative ISG (*MX1*) in protein lysates harvested from HIV-1-infected primary CD4+ T cells at day 5 post-infection in two biological replicates (donors A and B).

To better understand the role of CPSF6 in HIV-1 infection, we used CRISPR-Cas9 ribonucleoproteins (crRNPs) to knock-out *CPSF6* in primary CD4+ T cells from four independent donors (Fig. 1a). Briefly, bulk CD4+ T cells isolated from the peripheral blood of healthy human donors were activated and electroporated with crRNPs targeting our genes of interest. Two independent guide RNA targeting *CPSF6* were used in each experiment alongside a non-targeting negative control guide and two previously validated^16^ positive control guides targeting the early-acting host dependency factors CXCR4 and CYPA. Efficient knock-out of CYPA and CPSF6 in each donor was validated by immunoblotting (Fig. 1b and **Extended Data Fig. 1a**). There was no significant difference in percent viable cells between the non-targeting control and any of the knock-outs as measured by amine dye staining and flow cytometry four days post-electroporation (Fig. 1c). Daily monitoring of viable cell count by flow cytometry and/or by a luminescent ATP detection assay in three additional donors likewise revealed only modest differences in cell proliferation or ATP levels over time, with the *CPSF6* knock-out cells showing only slight decreases in each metric by ten days post-electroporation (**Extended Data Fig 1b**).

To determine the impact of *CPSF6* knock-out on HIV-1 infection, cells were subsequently challenged with replication-competent HIV-1 NL4–3 nef:IRES:GFP at 4 days post-electroporation in six technical replicates. The following day, three replicates were treated with the protease inhibitor Saquinavir (SQV, final concentration 5 μM) to limit infection to a single round of replication while the other three replicates were treated with a matched volume of DMSO as a vehicle control. Percent infected (GFP+) cells were monitored by flow cytometry at 2 and 5 days post-infection and normalized to the non-targeting control (Fig. 1d). As expected, *CXCR4* and *CYPA* knock-out strongly repressed infection at days 2 and 5, with the SQV-treated samples confirming their role in the early phase of the lifecycle. *CPSF6* knock-out, on the other hand, resulted in a 2- to 4-fold increase in infection at day 2, and a 3- to 9-fold increase in infection at day 5. In the presence of SQV, *CPSF6* knock-out cells maintained a 1.5- to 3-fold increase in infection at both days 2 and 5, suggesting that this increased permissivity is due, at least in part, to changes in the early stage of the lifecycle.

However, unlike knock-out of the *CXCR4* or *CYPA* dependency factors, which resulted in a consistent drop in infection over a 40-fold range of input virus, the effect of *CPSF6* knock-out was dose dependent (**Extended Data Fig. 1c**). Lower doses of virus resulted in the largest fold changes in infection relative to non-targeting at day 5 while the highest dose challenges resulted in no significant difference. This suggests that CPSF6 knock-out lowers a surmountable barrier to infection as opposed to facilitating a rate-limiting step that cannot be overcome by mass action.

Upon induction of the innate immune response, interferon (IFN) signaling results in the expression of a myriad of IFN-stimulated genes (ISGs), many of which have antiviral functions^34^. To determine if *CPSF6* knock-out could be altering the innate immune response to infection, we assessed expression of a representative ISG, MX1, in primary CD4+ T cells from the four independent donors above at 5 days post-infection (Fig. 1e). While MX1 expression varied by donor in the non-targeting cells, the *CYPA* knock-out cells had increased MX1 expression while the *CPSF6* knock-out cells had decreased in MX1 expression. Together, these results suggest that CPSF6 plays a role in modulating the innate immune response to HIV-1 infection in CD4+ T cells.

### *CPSF6* knock-out primary CD4+ T cells exhibit broad transcriptional reprogramming

**Figure 2:**
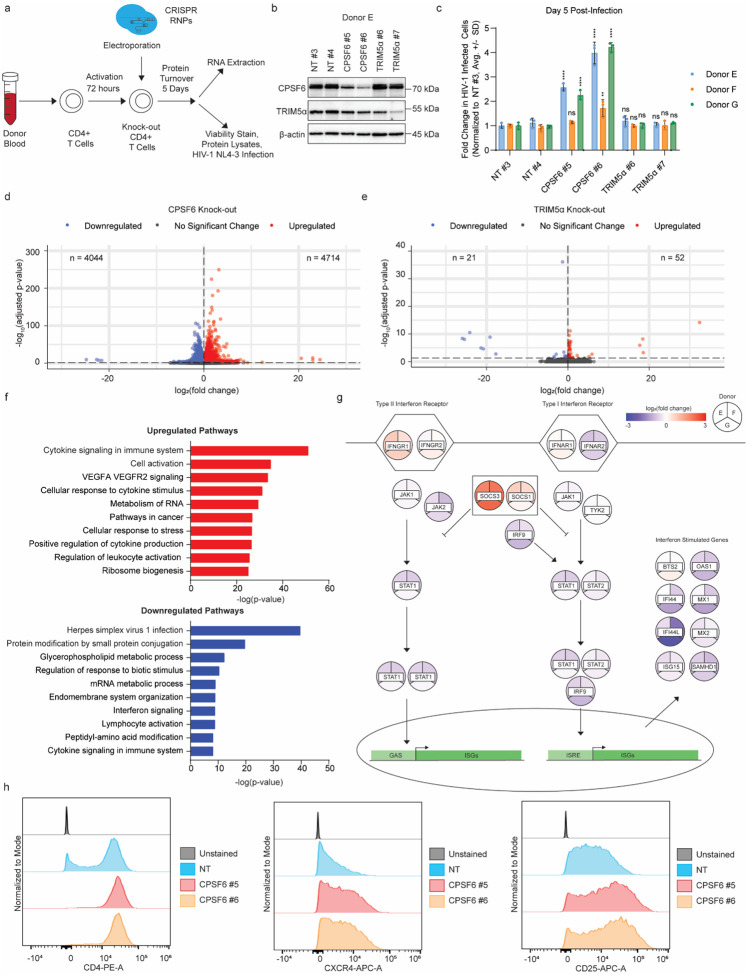
*CPSF6* knock-out primary CD4+ T cells exhibit broad transcriptional reprogramming. a, Schematic shows experimental workflow for RNA-sequencing. Primary CD4+ T cells were isolated from the blood of 3 independent donors and edited via electroporation of two crRNPs targeting CPSF6 and *TRIM5a* alongside non-targeting (NT) controls. RNA was extracted at day 5 post-electroporation and used for bulk RNA sequencing. In parallel, additional cells were used for viability assays, protein knock-out verification, and spreading infection assays. b, Immunoblot shows knock-out of *CPSF6* and *TRIM5α* in primary CD4+ T cell protein lysates harvested at day 5 post-editing in one representative biological replicate (donor E). c, HIV-1 infectivity (% GFP positive cells) at day 5 post-challenge with HIV-1 NL4–3 nef:IRES:GFP in indicated knock-out primary CD4+ T cells from 3 biological replicates (donors E-G) as assessed by flow cytometry. Each bar represents the average of technical triplicates +/− SD with individual data points shown. Statistics were calculated relative to the NT control per condition by one-way ANOVA with Dunnet’s test for multiple comparisons; ns = not significant, * = p ≤ 0.05, ** = p ≤ 0.01, *** = p ≤ 0.001, **** = p ≤ 0.0001. d, Volcano plot shows differentially expressed genes in RNA-Seq data from uninfected *CPSF6* knock-out primary CD4+ T cells as compared to NT controls in 3 biological replicates (donors E-G). Dots show significantly downregulated (blue) and upregulated (red) genes, dotted line shows threshold of 0.05 for adjusted p-value. e, Volcano plot shows differentially expressed genes in RNA-Seq data from uninfected *TRIM5α* knock-out primary CD4+ T cells as compared to NT controls in 3 biological replicates (donors E-G). Dots show significantly downregulated (blue) and upregulated (red) genes, dotted line shows threshold of 0.05 for adjusted p-value. f, Charts show functional enrichment (Metascape) analysis of top 10 upregulated (top, red) and downregulated (bottom, blue) pathways in differentially expressed genes from *CPSF6* knock-out primary CD4+ T cells as compared to NT controls in aggregate RNA-seq data from 3 biological replicates (donors E-G). g, Heatmap shows differential gene expression (log_2_(fold change)) for 3 biological replicates (donors E-G) in *CPSF6* knock-out primary CD4+ T cells as compared to NT controls overlayed on schematic of signaling events downstream of the type I and type II interferon receptors, including ISGs that are induced by these signaling pathways. h, Histograms show fluorescence intensity of CD4 (left), CXCR4 (middle), and CD25 (right) in primary CD4+ T cells from one representative biological replicate (donor I) at day five post-editing as measured by immunostaining and flow cytometry. Y-axis is normalized to the mode, histogram visualized using FlowJo version 10.10.

Given the observed downregulation of MX1 and the known role of CPSF6 in transcriptional processing, we hypothesized that *CPSF6* knock-out was regulating innate immune induction through transcriptional reprogramming. To explore this hypothesis, we performed bulk RNA-sequencing (RNA-Seq) on uninfected *CPSF6* knock-out primary CD4+ T cells from three independent donors (Fig. 2a). CPSF6 knock-out primary CD4+ T cells were generated via CRISPR-Cas9 gene editing as above alongside non-targeting and *TRIM5α* knock-out controls (2 independent guide RNA per condition). TRIM5α is an HIV-1 restriction factor that inhibits the early stage of the lifecycle through binding and premature disassembly of incoming cores^18,19^. It has been previously implicated in innate immune signaling upon core binding and oligomerization^35,36^. After allowing 5 days for protein turnover, RNA was extracted from the edited cells and processed for RNA-Seq. *CPSF6* and *TRIM5α* knock-out was confirmed in each donor by immunoblot (Fig. 2b, **Extended Data Fig. 2a**), and each cell population retained high viability (**Extended Data Fig. 2b**). These *CPSF6* knock-out cells exhibited enhanced permissivity to HIV-1 infection, in line with our previous observations (Fig. 1d), while knock-out of *TRIM5α* had no impact on wild-type infection as we previously reported^16^ (Fig. 2c).

Principal component analysis of the RNA-Seq data revealed that while the non-targeting and *TRIM5α* knock-out specimens clustered tightly together, the *CPSF6* knock-out specimens clustered separately (**Extended Data Fig. 2c**). After normalization to account for donor-to-donor variation, both guides from each donor were treated as replicates (n = 6 replicates per condition). Differential gene expression analysis revealed broad transcriptional reprogramming of the *CPSF6* knock-out cells as compared to non-targeting controls with 4,714 upregulated and 4,044 downregulated genes (Fig. 2d, **Extended Data Fig. 2d**). In contrast, the transcriptional profile of the *TRIM5α* knock-out cells remained nearly unchanged with only 52 upregulated and 21 downregulated genes (Fig. 2e, **Extended Data Fig. 2d**).

Functional enrichment analysis revealed that several genes involved in cytokine signaling and T cell activation were upregulated in the *CPSF6* knock-out cells (Fig. 2f), including *CD25* and *CXCR4*. To determine if these transcriptional changes were reflected in changes to cell surface expression, we repeated our knock-outs of *CPSF6* and *TRIM5α* using two independent guides in three additional donors alongside non-targeting, *CXCR4*-targeting, and *CYPA*-targeting controls. At 5 days post-editing, we performed cell surface immunostaining for the HIV-1 receptor CD4, co-receptors CXCR4 and CCR5, and activation marker CD25, quantifying mean fluorescence intensity (MFI) by flow cytometry. While *TRIM5α* knock-out had no significant impact on surface receptor expression, *CPSF6* knock-out cells displayed a slight, but significant increase in the cell surface expression of CD4 and larger increases in cell surface expression of CXCR4 and CD25 (Fig. 2h, **Extended Data Fig. 2f**). *CPSF6* knock-out did not have a significant impact on cell surface expression of CCR5 (**Extended Data Fig. 2g**). Thus, increased co-receptor expression and enhanced T cell activation may contribute to the enhanced HIV-1 replication observed in the *CPSF6* knock-out primary CD4+ T cells. To test this, we again repeated our knock-outs of *CPSF6* using two independent guides in three additional donors alongside non-targeting, *CXCR4*-targeting, and CYPA-targeting controls (validated by immunoblot, **Extended Data Fig. 2h**). Cells were subsequently challenged in technical triplicate with various p24-normalized amounts of either replication-competent HIV-1 NL4–3 nef:IRES:GFP or VSV-G pseudotyped, replication-incompetent HIV-1 NL4–3 dEnv nef:IRES:GFP at 4 days post-electroporation. Percent infected (GFP+) cells were monitored by flow cytometry at 2 days post-infection and normalized to the non-targeting control (**Extended Data Fig. 2i**). While the replication-competent virus again showed a 2- to 3-fold increase in replication in *CPSF6* knock-out cells, this effect was negated by VSV-G pseudotyping (as previously reported^24^), suggesting that enhanced co-receptor expression is at least in part responsible for the increased permissivity.

Several pathways involved in the innate immune response were simultaneously observed to be downregulated in *CPSF6* knock-out cells, including interferon signaling (Fig. 2f). Key components of the type I and type II interferon signaling pathway were identified as being downregulated in each donor, including *IFNAR2, STAT3, IRF9*, and *JAK2*, while negative regulators of the pathway were upregulated, including *SOCS1* and *SOCS3* (Fig. 2g and **Extended Data Fig. 2e**). Several ISGs and known antiretroviral restriction factors were also downregulated, including *SAMHD1* and *TRIM5α* (Fig. 2g and **Extended Data Fig. 2e**). Indeed, decreased TRIM5α protein levels were observed in all three donors upon *CPSF6* knock-out in the knock-out validation immunoblots (Fig. 2b and **Extended Data Fig. 2a**). These results suggest that *CPSF6* knock-out leads to broad transcriptional reprogramming in primary CD4+ T cells that results in upregulation of the viral receptors and downregulation of the innate immune response, ultimately resulting in increased permissivity to HIV-1 infection.

### The interferon response is dampened in *CPSF6* knock-out primary CD4+ T cells

**Figure 3:**
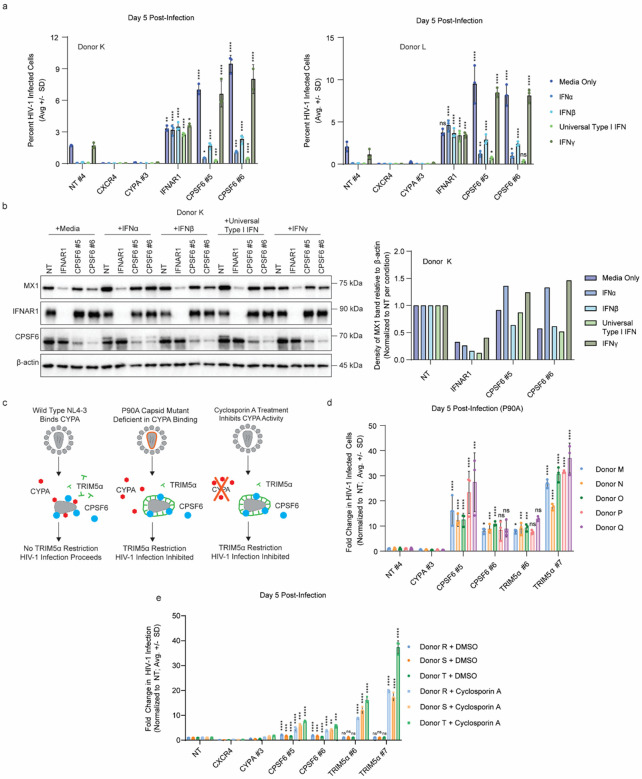
CPSF6 regulates the interferon pathway and expression of HIV-1 restriction factor TRIM5α in primary CD4+ T cells. a, HIV-1 infectivity (% GFP positive cells) at day 5 post-challenge with HIV-1 NL4–3 nef:IRES:GFP in indicated knock-out primary CD4+ T cells from 2 biological replicates (donors K and L) as assessed by flow cytometry. Cells were pre-treated with 100 U/mL IFNα, IFNβ, IFNγ, Universal Type I IFN, or media only control. Each bar represents the average of technical triplicates +/− SD with individual data points shown. Statistics were calculated relative to the non-targeting (NT) control per condition by one-way ANOVA with Dunnet’s test for multiple comparisons; * = p ≤ 0.05, ** = p ≤ 0.01, *** = p ≤ 0.001, **** = p ≤ 0.0001. b, Immunoblot shows expression of representative ISG (MX1) in knock-out primary CD4+ T cells treated with 10 U/mL IFNα, IFNβ, Universal Type I IFN, IFNγ, or media-only control for 16 hours in one representative biological replicate (donor K). Chart shows MX1 band density divided by β-actin band density in adjacent blot. Density measurements are normalized to NT controls per condition. c, Wild type HIV-1 NL4–3 capsid binds CYPA, which shields from TRIM5α restriction. The P90A capsid mutant HIV-1 derived from NL4.3 nef:IRES:GFP is deficient in CYPA binding, leading to TRIM5α-mediated restriction. Cyclosporin A (CsA) inhibits CYPA activity, leading to TRIM5α-mediated restriction. d, HIV-1 infectivity (% GFP positive cells) at day 5 post-challenge with HIV-1 NL4–3 nef:IRES:GFP P90A capsid mutant in indicated knock-out primary CD4+ T cells from 5 biological replicates (donors M-Q) as assessed by flow cytometry. Each bar represents the average of technical triplicates +/− SD with individual data points shown. Statistics were calculated relative to the NT control per condition by one-way ANOVA with Dunnet’s test for multiple comparisons; * = p ≤ 0.05, ** = p ≤ 0.01, *** = p ≤ 0.001, **** = p ≤ 0.0001.; * = p ≤ 0.05, ** = p ≤ 0.01, *** = p ≤ 0.001, **** = p ≤ 0.0001. e, HIV-1 infectivity (% GFP positive cells) at day 5 post-challenge with HIV-1 NL4–3 nef:IRES:GFP in indicated knock-out primary CD4+ T cells from 3 biological replicates (donors R-T) as assessed by flow cytometry. Cells were pre-treated with cyclosporin A or a DMSO control 4 hours prior to infection. Each bar represents the average of technical triplicates +/− SD with individual data points shown. Statistics were calculated relative to the NT control per condition by one-way ANOVA with Dunnet’s test for multiple comparisons; * = p ≤ 0.05, ** = p ≤ 0.01, *** = p ≤ 0.001, **** = p ≤ 0.0001.; * = p ≤ 0.05, ** = p ≤ 0.01, *** = p ≤ 0.001, **** = p ≤ 0.0001.

Transcriptional downregulation of the interferon signaling pathway in *CPSF6* knock-out cells suggests one mechanism for their enhanced permissivity. To test this directly, primary CD4+ T cells from three independent donors were electroporated with two independent crRNPs targeting *CPSF6* alongside non-targeting, *CXCR4*-targeting, and *CYPA*-targeting controls as well as a multiplexed pool of five crRNPs targeting the type I interferon receptor gene (*IFNAR1*). Cells were then pre-treated with media alone or 10 U/mL IFNα, IFNβ, Universal type I IFN, or IFNγ for 16 hours prior to challenge with replication-competent HIV-1 NL4–3 nef:IRES:GFP. At 5 days post-challenge, in the media only condition, *CXCR4* and *CYPA* knock-out decreased infection while *CPSF6* increased infection as expected (Fig. 3a, **Extended Data Fig. 3a**). Knock-out of *IFNAR1* resulted in a roughly two-fold increase in infection over the non-targeting control compared to the 4- to 5-fold increase observed in the *CPSF6* knock-out cells. IFNα, IFNβ, and Universal type I IFN treatment decreased infection in the non-targeting cells while IFNγ had no significant impact. The *IFNAR1* knock-out cells were not impacted by IFN treatment as expected. Notably, *CPSF6* knock-out cells showed a dampened response to IFNα, IFNβ, and Universal type I IFN treatment with a partial rescue of HIV-1 replication in these cells. Protein lysates collected after interferon pre-treatment, but prior to infection, showed decreased levels of a representative ISG, MX1, upon *CPSF6* knock-out, though not to the same extent as observed upon *IFNAR1* knock-out (Fig. 3b, **Extended Data Fig. 3b**). Taken together, these data suggest that reduced IFN signaling and responsiveness in part explain the enhanced permissivity of *CPSF6* knock-out cells to HIV-1 infection.

The RNA-Seq data also showed downregulation of a number of innate immune effectors and known HIV-1 restriction factors upon *CPSF6* knockout, including TRIM5α, which binds to and restricts incoming cores. To protect itself from TRIM5α-mediated restriction, incoming cores bind CYPA, which masks the TRIM5α binding site^15,16^. Disruption of CYPA binding either by mutation of the capsid *(i.e*., through a capsid P90A mutation) or through treatment with the CYPA inhibitor cyclosporin A (CsA) decreases HIV-1 infection due to TRIM5α-mediated restriction (Fig. 3c). To determine if *CPSF6* knock-out cells were functionally defective in TRIM5α, we electroporated primary CD4+ T cells from five independent donors with two independent crRNPs targeting *CYPA, CPSF6*, or *TRIM5α* alongside a non-targeting control (immunoblot validation in **Extended Data Fig. 3c**). Cells were then challenged with HIV-1 NL4–3 nef:IRES:GFP containing a P90A capsid mutant in technical triplicate. Across all donors, *CYPA* knock-out had no significant effect on P90A virus infection, consistent with the inability of P90A capsids to recruit CYPA. Knock-out of *TRIM5α* led to a 10- to 30- fold increase in infection correlating with knock-out efficiency as assessed by immunoblot (**Extended Data Fig. 3c**), consistent with prior reports^16^. Knock-out of *CPSF6* led to a decrease in TRIM5α steady-state levels across all donors (**Extended Data Fig. 3c**) and rescued P90A virus replication to similar levels of as *TRIM5α* knock-out itself (Fig. 3d).

To further confirm these findings, we knocked out *CPSF6* and *TRIM5α* using two independent guides in three additional donors alongside non-targeting, *CXCR4*-targeting, and *CYPA*-targeting controls (immunoblot validation in **Extended Data Fig. 3d**). Each population was challenged with HIV-1 NL4–3 nef:IRES:GFP in the presence and absence of CsA in technical triplicate. In the absence of CsA, *CYPA* knock-out decreased infection, *CPSF6* knock-out increased infection, and *TRIM5α* knock-out had no effect (Fig. 3e). CsA strongly inhibited replication in the non-targeting cells, though this was rescued in the *TRIM5α* knock-out cells with the degree of rescue correlating with knock-out efficiency (**Extended Data Fig. 3d**). CPSF6 knock-out partially rescued replication in the presence of CsA, consistent with the P90A capsid mutant data. These data suggest that the broad transcriptional reprogramming upon *CPSF6* knock-out leads to decreased interferon signaling and reduced restriction factor expression, both of which partially contribute to enhanced HIV-1 replication in these cells.

### *CPSF6* knock-out in primary CD4+ T cells leads to changes in alternative polyadenylation

**Figure 4:**
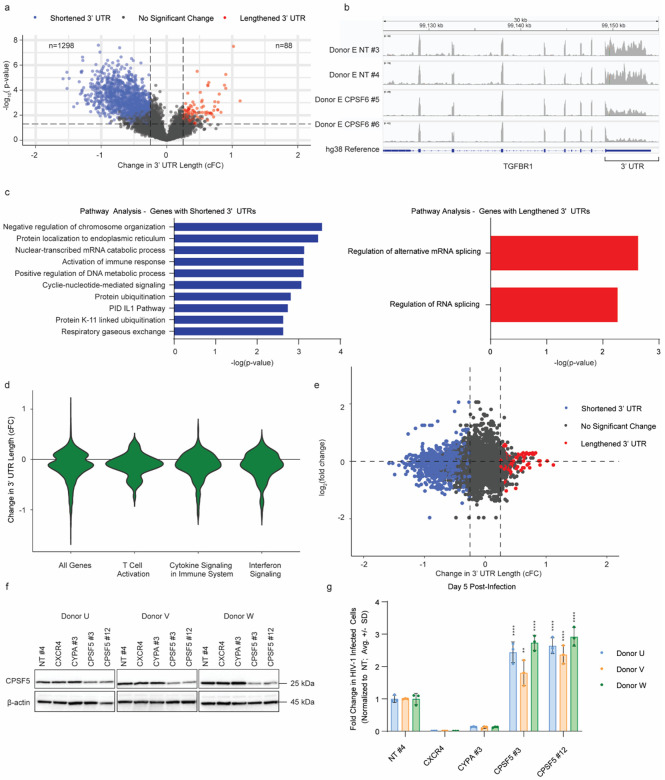
CPSF6 knock-out leads to changes in alternative polyadenylation in primary CD4+ T cells a, Volcano plot shows REPAC analysis of changes in 3’ UTR lengths for *CPSF6* knock-out primary CD4+ T cells as compared to non-targeting (NT) controls in 3 biological replicates (donors E-G, RNA-seq data from Fig.2). 3’ UTRs are designated shortened (blue) for Compositional Fold Change (cFC) < 0.25 and −log_10_(adjusted p-value) < 0.05 and lengthened (red) for Compositional Fold Change (cFC) > 0.25 and −log_10_(adjusted p-value) < 0.05. Dots represent individual tested poly(A) sites. b, Tracks show read coverage of TGFBR1 in RNA-seq data from *CPSF6* knock-out and NT control cells from one representative biological replicate (donor E), visualized using Integrative Genomics Visualizer. 3’ UTRs show evidence of shortening in response to *CPSF6* knock-out. c, Charts show functional enrichment (Metascape) analysis of top 10 pathways enriched in genes with shortened 3’ UTRs (left, blue) and lengthened 3’ UTRs (right, red) in response to *CSPF6* knock-out in primary CD4+ T cells as assessed by REPAC analysis in 3 biological replicates (donors E-G). d, Violin plot shows comparison of cFC values from REPAC analysis in all tested genes as compared to genes in the indicated gene sets (GO:0042110: T Cell activation, R-HSA-1280215: Cytokine Signaling in Immune system, R-HSA-913531: Interferon Signaling) in 3 biological replicates (donors E-G). e, Scatterplot shows Compositional Fold Change (cFC) of all tested APA sites in REPAC analysis of *CPSF6* knock-out primary CD4+ T cells in as compared to NT controls plotted against differential expression data as shown in Fig.2 in data aggregated from three biological replicates (donors E-G). 3’ UTRs are designated shortened (blue) for Compositional Fold Change (cFC) < 0.25 and −log_10_(adjusted p-value) < 0.05 and lengthened (red) for Compositional Fold Change (cFC) > 0.25 and −log_10_(adjusted p-value) < 0.05. Dots represent individual tested poly(A) sites. f, Immunoblot shows knock-out of *CPSF5* in protein lysates harvested at day 4 post-editing in 3 biological replicates (donor U-W). g, HIV-1 infectivity (% GFP positive cells) at day 5 post-challenge with HIV-1 NL4–3 nef:IRES:GFP in indicated knock-out primary CD4+ T cells from 3 biological replicates (donors U-W) as assessed by flow cytometry. Each bar represents the average of technical triplicates +/− SD with individual data points shown. Statistics were calculated relative to the NT control per condition by one-way ANOVA with Dunnet’s test for multiple comparisons; * = p ≤ 0.05, ** = p ≤ 0.01, *** = p ≤ 0.001, **** = p ≤ 0.0001.

Given that CPSF6 regulates polyadenylation site selection through its role in the CFIm complex, we hypothesized that the broad transcriptional changes in primary CD4+ T cells upon *CPSF6* knock-out could be driven by changes in APA. To test this hypothesis, we re-analyzed our RNA-seq data from uninfected *CPSF6* knock-out primary CD4+ T cells using regression of polyadenylation compositions (REPAC) analysis^37^. Briefly, this algorithm compares RNA-seq reads on either side of known, annotated poly(A) sites and calculates the Compositional Fold Change (cFC) for each gene as a measure of 3’UTR length changes between an experimental *(CPSF6* knock-out) and control (non-targeting) condition. REPAC analysis revealed that *CPSF6* knock-out primary CD4+ T cells exhibited an overall shortening of 3’UTR lengths (n = 1,298 poly(A) sites) as compared to non-targeting controls, consistent with what has been observed in other cell types^11^ (Fig. 4a). These changes in 3’ UTR lengths can be readily observed in the transcript read coverage tracks, as shown at the transforming growth factor-beta receptor type 1 (TGFBR1) locus, for example (Fig. 4b). Functional enrichment analysis of genes with significantly shortened 3’UTRs identified broad changes in APA at genes associated with immune response signaling, suggesting that shortening of these transcripts may be associated with their downregulation (Fig. 4c). To validate this result, we plotted the cFC values for all genes previously identified in the functional enrichment analysis associated with the term ‘Interferon Signaling’ (Fig. 2e) and found these genes also showed evidence of 3’ UTR shortening (Fig. 4d).

A decrease in 3’UTR length was not universally associated with decreased transcript abundance. Indeed, similar decreases in cFCs were observed for transcripts associated with the ‘Cytokine Signaling in Innate Immune Signaling’ and ‘T Cell Activation’ pathways, both of which were enriched pathways in the upregulated genes (Fig. 4d, Fig. 2f). To assess the relationship between changes in 3’ UTR length and transcript abundance more globally, we plotted cFC values from all analyzed poly(A)sites against the associated log_2_(fold change) values from the differential gene expression analysis in Fig. 2. While there was a bias towards genes with shortened 3’ UTRs exhibiting downregulation, changes in 3’ UTR length were associated with variable changes in transcript abundance, which suggests multiple modes of gene-dependent regulation (Fig. 4e). Additionally, several genes with significant changes in abundance did not exhibit a significant change in 3’UTR length, suggesting there are additional and/or secondary effects of *CPSF6* knock-out on the transcriptome by this timepoint (Fig. 4e). *CXCR4* and *CD25*, for example, were upregulated and had increased cell surface expression (Fig. 2f), but only *CD25* showed a significant decrease in 3’UTR length (**Extended Data Fig. 4a**).

Several different algorithms have been developed to assess poly(A) site usage, all of which use slightly different approaches. The REPAC algorithm uses only well-annotated poly(A) sites, but as a result does not give information on every locus. Therefore, to validate these results across the transcriptome, we leveraged an alternative method of APA analysis that infers poly(A) site usage and calculates changes in the distal polyadenylation usage index (ΔPDUI) as a proxy for changes in 3’UTR length for each gene (**Extended Data Fig. 4b**). As with the REPAC analysis, ΔPDUI analysis showed that *CPSF6* knock-out led to global 3’ UTR shortening, with genes involved in the interferon pathway, cytokine signaling, and cellular activation showing preferential 3’ UTR shortening (**Extended Data Fig. 4c**).

These data suggest that changes in APA are induced by *CPSF6* knock-out and are at least partially responsible for the broad transcriptional reprogramming and increased permissivity to HIV-1 observed in these cells. If this is true, we would hypothesize that knock-out of the other CFIm complex member, CPSF5, would result in a similar phenotype. To test this hypothesis, we knocked out *CPSF5* in primary CD4+ T cells using two independent guide RNA in three independent donors and conducted HIV-1 spreading infection assays as before alongside non-targeting, *CXCR4* knock-out, and *CYPA* knock-out controls. *CPSF5* knock-out had minimal impact on cell viability (**Extended Data Fig. 4d**). While CPSF5 knock-out efficiency wasn’t as high as that achieved for CPSF6 as monitored by immunoblot (Fig. 4f), it still resulted in a significant increase in HIV-1 infection (Fig. 4g). Taken together, these data suggest that the induction of APA through CFIm complex disruption results in broad transcriptional reprogramming and enhanced CD4+ T cell permissivity to HIV-1 infection.

### HIV-1 capsid binding to CPSF6 triggers transcriptional rewiring

Previously described roles of CPSF6 in HIV-1 infection, including its roles in nuclear import and integration site selection, are dependent upon CPSF6 binding to HIV-1 cores. Several studies have shown that this interaction can lead to relocalization of CPSF6 to nuclear speckles^38–41^. However, it is unclear if this interaction and the subsequent relocalization of CPSF6 may be sufficient to disrupt CFIm function and trigger changes in APA. To determine whether HIV-1 infection induces APA and transcriptional reprogramming through recruitment of CPSF6 to capsid cores, we challenged primary CD4+ T cells from three independent donors with HIV-1 NL4–3 nef:IRES:GFP encoding either wild-type (WT) capsid or a capsid mutant virus that is deficient in CPSF6 binding (N74D). At day 2 post-challenge, infected (GFP+) cells were sorted to obtain pure populations of infected cells, alongside uninfected controls, from which RNA was extracted and submitted for RNA-Seq (**Extended Data Fig. 5a**). In parallel, we generated *CPSF6* knock-out and non-targeting control primary CD4+ T cells and extracted RNA at 2 days post-electroporation to directly compare the transcriptional profiles between the infected cells at day 2 post-infection and *CPSF6* knock-out cells at day 2 post-editing (immunoblot validation of knock-out efficiency in **Extended Data Fig. 5b**).

**Figure 5:**
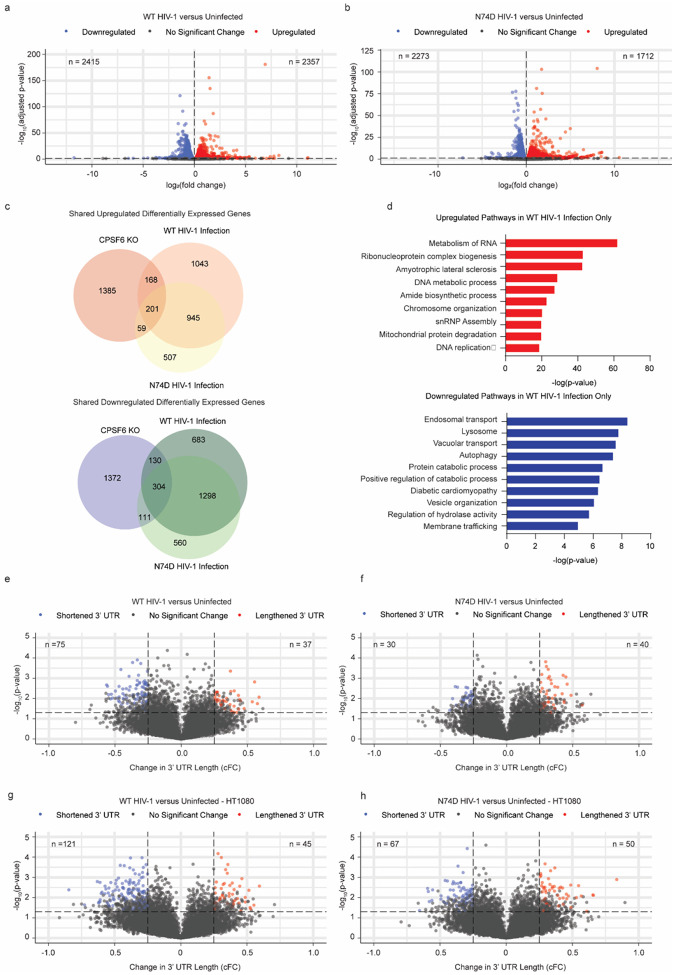
HIV-1 infection induces APA in primary CD4+ T cells. a, Volcano plot shows differentially expressed genes in RNA-Seq data from wild-type (WT) HIV-1 NL4–3 nef:IRES:GFP-infected primary CD4+ T cells as compared to uninfected controls in 3 biological replicates (donors X-Z). Dots show significantly downregulated (blue) and upregulated (red) genes, dotted line shows threshold of 0.05 for adjusted p-value. b, Volcano plot shows differentially expressed genes in RNA-seq data from N74D capsid mutant HIV-1 NL4–3 nef:IRES:GFP-infected primary CD4+ T cells as compared to uninfected controls in 3 biological replicates (donors X-Z). Dots show significantly downregulated (blue) and upregulated (red) genes, dotted line shows threshold of 0.05 for adjusted p-value. c, Venn diagrams shows overlap of upregulated (top) and downregulated (bottom) differentially expressed genes between *CPSF6* knock-out, WT infected, and N74D infected primary CD4+ T cells in 3 biological replicates (donors X-Z). d, Charts show functional enrichment (Metascape) analysis of top 10 upregulated (top, red) and downregulated (bottom, blue) pathways in differentially expressed genes from WT HIV-1 NL4–3 infected cells that are not differentially expressed in N74D HIV-1 NL4–3 infected cells at day 2 post-infection in 3 biological replicates (donors X-Z). e, Volcano plot shows REPAC analysis of changes in 3’ UTR lengths for WT HIV-1 NL4–3 nef:IRES:GFP infected versus uninfected primary CD4+ T cells from 3 biological replicates (donors X-Z). 3’ UTRs are designated shortened (blue) for Compositional Fold Change (cFC) < 0.25 and −log_10_(adjusted p-value) < 0.05 and lengthened (red) for Compositional Fold Change (cFC) > 0.25 and −log_10_(adjusted p-value) < 0.05. Dots represent individual tested poly(A) sites, f, Volcano plot shows REPAC analysis of changes in 3’ UTR lengths for N74D capsid mutant HIV-1 NL4–3 nef:IRES:GFP infected versus uninfected primary CD4+ T cells from 3 biological replicates (donors X-Z). 3’ UTRs are designated shortened (blue) for Compositional Fold Change (cFC) < 0.25 and −log_10_(adjusted p-value) < 0.05 and lengthened (red) for Compositional Fold Change (cFC) > 0.25 and −log_10_(adjusted p-value) < 0.05. Dots represent individual tested poly(A) sites, f, Volcano plot shows REPAC analysis of changes in 3’ UTR lengths for WT VSV-G-pseudotyped HIV-1-NL4–3-infected versus uninfected HT1080 cells from 3 technical replicates. Cells were infected at an MOI of 10 and RNA was extracted at 6 hours post-infection. 3’ UTRs are designated shortened (blue) for Compositional Fold Change (cFC) < 0.25 and −log_10_(adjusted p-value) < 0.05 and lengthened (red) for Compositional Fold Change (cFC) > 0.25 and −log_10_(adjusted p-value) < 0.05. Dots represent individual tested Poly(A) sites. g, Volcano plot shows REPAC analysis of changes in 3’ UTR lengths for N74D VSV-G-pseudotyped HIV-1-NL4–3-infected versus uninfected HT1080 cells from 3 technical replicates. Cells were infected at an MOI of 10 and RNA was extracted 6 hours post-infection. 3’ UTRs are designated shortened (blue) for Compositional Fold Change (cFC) < 0.25 and −log_10_(adjusted p-value) < 0.05 and lengthened (red) for Compositional Fold Change (cFC) > 0.25 and −log_10_(adjusted p-value) < 0.05. Dots represent individual tested poly(A) sites.

Similar to what was reported earlier, *CPSF6* knock-out resulted in broad transcriptional rewiring of primary CD4+ T cells with 3,730 differentially expressed genes, including upregulation of genes involved in cytokine signaling and downregulation of those involved in the innate immune response (**Extended Data Fig. 5c** and **5d**). Infection with WT virus likewise resulted in broad transcriptional changes with 4,772 differentially expressed genes (Fig. 5a). Infection with the N74D virus, on the other hand, resulted in slightly less transcriptional rewiring, with 3,985 differentially expressed genes (Fig. 5b). Comparing between the conditions, the WT infected and *CPSF6* knock-out cells shared 803 misregulated genes, (Fig. 5c, **Extended Data Fig. 5e**), and the N74D infected and *CPSF6* knock-out cells shared 675 misregulated genes (Fig. 5c). Functional enrichment analysis revealed that genes involved in mRNA metabolism and endosomal transport were enriched in the fraction of differentially expressed genes that were found in the WT infected, but not the N74D infected condition (Fig. 5d).

These data suggest that the interaction between CPSF6 and HIV-1 capsid cores may be responsible for some of the transcriptional reprogramming observed upon infection. To assess if these changes were associated with changes in APA, we next performed REPAC analysis on the RNA-Seq data from the infected cells. The *CPSF6* knock-out cells, as before, showed an overall shortening of 3’UTR lengths with 362 poly(A) sites and 22 poly(A) sites exhibiting 3’UTR lengthening and shortening, respectively (**Extended Data Fig. 5f**). The primary T cells infected with WT virus showed a similar bias towards an overall shortening of 3’UTR lengths as compared to uninfected controls, though at fewer loci (37 lengthened, 75 shortened; Fig. 5e). On the contrary, the cells infected with the N74D virus did not show a directional bias in 3’UTR lengths as compared to uninfected controls (40 lengthened, 30 shortened; Fig. 5f). These data were independently confirmed using the ΔPDUI algorithm, which likewise confirmed a bias towards 3’ UTR shortening in the WT virus infected cells, but not the N74D virus infected cells (**Extended Data Fig. 5g** and **5h**). Similar to the *CPSF6* knock-out data, a majority of gene expression changes cannot be directly linked to a change in 3’UTR length at this timepoint, though this doesn’t rule out secondary or indirect effects of APA.

We next examined whether changes in CPSF6 localization led to disruption of CPSF6 function and changes in APA in a cell line model of HIV-1 infection. Recruitment of CPSF6 to nuclear speckles has been observed during HIV-1 infection, with the extent of colocalization between CPSF6 and nuclear speckles dependent on cell type and timepoint^38–41^. Furthermore, this phenotype may be dependent on CPSF6 binding of HIV-1 cores, and infection with the N74D capsid mutant virus does not lead to colocalization of CPSF6 with markers of nuclear speckles in some cell types^41^. A recent study observed colocalization of CPSF6 with nuclear speckles in primary CD4+ T cells even in the absence of HIV-1 infection, indicating that CPSF6 may not undergo major changes in localization following infection of this cell type^42^. Given that wild-type HIV-1 infection does not induce changes in APA to the same degree as *CPSF6* knock-out in primary CD4+ T cells, we wanted to determine whether cell lines that undergo more significant changes in CPSF6 localization following HIV-1 infection exhibit greater disruption of CPSF6 function and changes in 3’ UTR length. We used HT1080 cells as a cell line model of CPSF6 recruitment to nuclear speckles, as recruitment of CPSF6 to nuclear speckles following HIV-1 infection was observed in 30–40% of infected HT1080 cells in prior studies^39^. To track changes in CPSF6 localization following HIV-1 infection, we engineered endogenous monomeric NeonGreen (mNGreen) C-terminal tagged CPSF6 into HT1080 cells (HT1080-CPSF6-mNGreen) via CRISPR-Cas9 knock-in. We infected HT1080-CPSF6-mNGreen cells with VSV-G pseudotyped-HIV-1 NL4–3 encoding either WT capsid or N74D capsid at a multiplicity of infection (MOI) of 10, then extracted RNA at 6 hours post infection and performed bulk RNA-Seq. In line with our primary cell data, REPAC analysis of the RNA-Seq data showed that WT infected HT1080 cells demonstrated a bias towards an overall shortening of 3’UTR lengths as compared to mock-infected controls (45 lengthened, 121 shortened; Fig. 5g), representing a more robust change in APA than what was seen in primary CD4+ T cells (Fig. 5e). Furthermore, HT1080 cells infected with N74D virus did not show a directional bias in changes to 3’UTR lengths as compared to mock-infected controls (50 lengthened, 67 shortened; Fig. 5h). Widefield deconvolution fluorescent microscopy imaging confirmed that infection with viruses with WT capsid led to formation of CPSF6 puncta in cell nuclei, and these puncta were not present in the mock or N74D infected cells (**Extended Data Fig. 5i**). These results suggest that changes in CPSF6 localization may disrupt CPSF6 function and induce transcriptional rewiring through changes to APA.

If transcriptional rewiring triggered by CPSF6 recruitment to capsid cores enhances CD4+ T cell permissivity to infection, we would expect that mutations that prevent CPSF6 binding would decrease infectivity and that knock-out of *CPSF6* would help rescue infection of those viruses. To test this, we electroporated primary CD4+ T cells from five independent donors with two independent crRNPs targeting *CPSF6* and *TRIM5α* alongside a non-targeting and CYPA-targeting control (immunoblot validation in Extended Data Fig. 3c). Cells were then challenged with HIV-1 NL4–3 nef:IRES:GFP containing the N74D capsid mutant in technical triplicate. At day 5 post-challenge, *CPSF6* knock-out cells showed a 4- to 6-fold increase in infection relative to the non-targeting controls (**Extended Data Fig. 5j**). Previous reports have shown the N74D capsid mutant also results in reduced binding of CYPA^24^ and knock-out of *TRIM5α* was sufficient to rescue infection to similar levels (**Extended Data Fig. 5j**). The pleiotropic nature of the N74D capsid mutant and the previously demonstrated impact of CPSF6 on TRIM5α levels complicates interpretation of this experiment. Nevertheless, these data are consistent with a model in which CPSF6 recruitment and relocalization by incoming capsid cores triggers transcriptional reprogramming of infected cells through APA to enhance permissivity to infection.

## DISCUSSION

The known functions of CPSF6 in nuclear import and integration site selection are not sufficient to explain the enhanced permissivity to HIV-1 infection observed upon *CPSF6* knock-out in primary CD4 + T cells. While the magnitude of this effect varies slightly by donor, we observed increased HIV-1 NL4–3 infection upon *CPSF6* knock-out in all 37 independent donors used in this study. Smaller increases in infection upon *CPSF6* knock-down have also been observed in other cell types, such as HEK293T and HeLa cells^11,23^. We propose a model wherein CPSF6 perturbation results in broad transcriptional reprogramming and enhanced permissivity to infection, which we demonstrate is mediated in part by enhanced receptor expression and a dampened interferon response. Given this multifaceted mechanism-of-action, we would expect this phenotype to be highly dependent on experimental set up with the cell type, viral tropism, and baseline immune activation all influencing the magnitude of the effect. It remains to be seen if there are additional cell type dependencies in the subsets of genes regulated by CPSF6 and what other host factors may dictate this selectivity.

One of the well-known functions of CPSF6 is its role in polyadenylation site selection as part of the CFIm complex with CPSF5. Prior studies have demonstrated that disruption of this complex results in changes in APA and an overall shortening of 3’UTR lengths^12,13^. Consistent with this, we observe a strong bias towards 3’UTR shortening in primary CD4 + T cells upon *CPSF6* knock-out using two independent algorithms. While the effect of 3’UTR length on transcript abundance was variable, genes with shortened 3’UTRs were generally downregulated. Genes in the interferon signaling pathway, for example, had preferentially shortened 3’ UTRs and were generally downregulated. This is in line with previous studies that have reported a role for APA in the regulation of the innate immune response to infection with other viruses, including Sendai Virus, Influenza A Virus, and Vesicular Stomatitis Virus^33,42,43^. CFIm complex disruption through knock-out of *CPSF5* also led to enhanced permissivity to HIV-1, suggesting that APA induction is responsible for the enhanced permissivity. That being said, the number of differentially expressed genes was substantially larger than the number of genes with altered 3’UTR lengths, suggesting alternate, indirect means by which *CPSF6* knock-out may lead to transcriptional reprogramming.

Incoming HIV-1 capsid cores are known to bind CPSF6 at a conserved interface with defined roles in nuclear entry and integration site selection^44^. Furthermore, in some cell types, HIV-1 infection has been shown to induce relocalization of nuclear CPSF6 to sites of pre-mRNA processing known as nuclear speckles^38–41^. We reasoned that if CPSF6 binding to the capsid and/or relocalization was sufficient to induce changes in APA, it could represent a novel mechanism by which the virus reprograms its target cells to optimize its replication. Indeed, we found that in HT1080 cells, which are reported to exhibit relocalization of CPSF6 to nuclear speckles following HIV-1 infection^39^, there is a bias towards 3’UTR shortening for wild-type infected cells, but not N74D infected cells at 6 hours post-infection. Furthermore, infection of primary CD4 + T cells with wild-type HIV-1 virus recapitulated many of the gene expression changes observed upon *CPSF6* knock-out, while infection with an N74D capsid mutant virus recapitulated fewer of these changes. We also observed a bias towards 3’UTR shortening in the wild-type infected primary CD4 + T cells, but not the N74D infected cells. That being said, the number of genes with significantly altered 3’UTR lengths was much smaller in infected primary CD4 + T cells compared to *CPSF6* knock-out cells or HIV-1 infected HT1080 cells. A recent study observed colocalization of CPSF6 with nuclear speckles in primary CD4 + T cells even in the absence of HIV-1 infection, indicating that CPSF6 may not undergo major changes in localization following infection of this cell type^45^. This could explain why we observed fewer changes in 3’ UTR lengths following wild-type HIV-1 infection in primary CD4 + T cells as compared to HT1080 cells. The degree of CPSF6 relocalization after infection varies by time and cell type; it remains to be seen to what extent these factors correlate with changes in polyadenylation.

CPSF6 binding to incoming capsid cores has been previously suggested to shield the incoming virus from innate immune sensing in myeloid cells^22^. While our models differ, these data are highly complementary to our observations, with the critical difference being that knock-down of *CPSF6* in primary macrophages was observed to inhibit HIV-1 replication. This is consistent with data in several other cell lines wherein CPSF6 depletion results in decreased viral replication associated with defects in nuclear import. Our data suggests that CPSF6 is not required for nuclear import in primary CD4 + T cells, though it remains to be seen if this is due to cell cycling or the role of some other redundant factor. In cell types where CPSF6 is required for nuclear import, we would expect this phenotype to be dominant, but this is not inconsistent with an additional role for CPSF6 recruitment in transcriptional reprogramming.

Taken together, our data suggest a model in which CPSF6 recruitment by incoming capsid cores triggers transcriptional reprogramming of infected cells through APA to enhance permissivity to infection. This reprogramming may reflect a means by which HIV-1 achieves a targeted host shutoff to dampen innate immune sensing and promote viral replication. Future studies will focus on understanding the viral and host determinants that govern the multifaceted role of CPSF6 in HIV-1 infection.

## METHODS

### Cell Lines

Human embryonic kidney (HEK) 293T cells were obtained from ATCC (#CRL-3216) and cultured in 1x Dulbecco’s Modified Eagle Medium (DMEM) (Corning, #10–013-CV) supplemented with 10% heat inactivated fetal bovine serum (Gibco, #16140–071) and 1% penicillin/streptomycin (Cytiva, #SV30010). 293AAV (Cell Biolabs, Cat# AAV-100) and HT1080 (ATCC, #CVCL_0317) cell lines were maintained in 1x DMEM (ThermoFisher, 11995065), supplemented with 10% fetal calf serum (Sigma, #F092) and gentamicin (Gibco, #15750060) Cells were maintained in a humidified cell culture incubator at 37°C with 5% CO_2_.

### Plasmid Constructs

An HIV-1 NL4–3 molecular clone with GFP cloned behind an IRES cassette following the viral *nef* gene (nef:IRES:GFP) was used as the base vector for all viral plasmids (NIH AIDS Reagent Program, #11349). N74D and P90A capsid mutants were generated from the original plasmid using site-directed mutagenesis (SDM). Briefly, a portion of the original NL4–3 nef:IRES:GFP capsid region between the SfoI and SpeI restriction sites was amplified and cloned into a pJet1.2/blunt vector (Thermo Scientific, #K1231). The pJet1.2 plasmid with the capsid insert was used for SDM to make the desired point mutations with the following primers: HIV-1 Capsid SDM N74D Forward (5’-GTTAAAAGAGACCATCGATGAGGAAGCTGCAG-3’), HIV-1 Capsid SDM N74D Reverse (5’-CTGCAGCTTCCTCATCGATGGTCTCTTTTAAC-3’), HIV-1 Capsid SDM P90A Forward (5’-TCCAGTGCATGCAGGGGCTATTGCACCAGGC-3’), HIV-1 Capsid SDM P90A Reverse (5’-GCCTGGTGCAATAGCCCCTGCATGCACTGGA-3’).

The PCR reactions were transformed into chemically competent *E. coli* (Takara Bio, #636763), and colonies were screened for the desired mutation by Sanger sequencing using the following primer sequences: 5’-CGACTCACTATAGGGAGAGCGGC-3’, 5’-AAGAACATCGATTTTCCATGGCAG-3’. Capsid region inserts bearing the desired mutation were isolated from the pJet1.2 vector and re-inserted into the original NL4–3 nef:IRES:GFP vector by Gibson assembly. Final plasmid sequences were confirmed via Sanger sequencing with the following primers: 5’-AGCGTCGGTATTAAGCGGGG-3’, 5’-ATTCCCTGGCCTTCCCTTGT-3’.

### Primary CD4+ T Cell Isolation and Culture

Primary CD4+ T cells were isolated from human peripheral blood leukopaks sourced from de-identified healthy donors (STEMCELL Technologies, #200–0092). Leukopak blood products were diluted to 150 mL in Dulbecco’s Phosphate-Buffered Saline (DPBS) (Corning, #21–031-CV) with 2mM EDTA (Corning, #46–034-Cl) per donor. PBMCs were isolated through Ficoll-Paque centrifugation (Cytiva, #17-5442-03) followed by washing with DPBS with 2mM EDTA. PBMCs were resuspended in MACS buffer (DPBS with 0.5% bovine serum albumin (BSA) (Fisher Scientific, #BP9706100), and 2mM EDTA), and CD4+ T cells were then isolated from PBMCs using an EasySep Human CD4+ T Cell Isolation Kit (STEMCELL Technologies, #17952) per the manufacturer protocol. Cells were resuspended at 2.5×10^6^ cells/mL in complete Roswell Park Memorial Institute (RPMI) medium consisting of RPMI 1640 (Corning, #10–040-CV) supplemented with 10% heat inactivated fetal bovine serum (Gibco, #16140–071), 12.5 mL HEPES (Cytiva, #SH30237.01), 5 mL sodium pyruvate (Corning, #25–000-CI), and 1% penicillin/streptomycin (Cytiva, #SV30010). Media was supplemented with interleukin-2 (IL-2) at 20 IU/mL immediately before use (Miltenyi, #130097744). Cells were activated on α-CD3-coated plates (Tonbo Biosciences, UCHT1) incubated at 4°C overnight before use, with soluble α-CD28 added at 5 mg/mL to media before plating cells (Tonbo Biosciences, CD28.2). Cells were stimulated for 72 hours at 37°C with 5% CO_2_ prior to downstream applications.

5–10×10^4^ PBMCs, unstimulated CD4+ T cells, and stimulated CD4+ T cells were taken for CD4 and CD25 cell surface marker staining (Miltenyi Biotec, #130-113-225, 1130-115-535, respectively) to assess cell population purity and activation for each donor. Stained samples were fixed in 1% formaldehyde in DPBS for analysis by flow cytometry within 1 week post-fixation.

### CRISPR-Cas9 Knock-outs in Primary CD4+ T cells

CRISPR-Cas9 knock-outs were generated following previously published protocols^46^. To synthesize CRISPR-Cas9 ribonucleoprotein complexes (crRNPs), lyophilized crRNA and tracrRNA (Dharmacon) were spun down and resuspended at 160 μM in buffer of 10 mM Tris-HCl (7.4 pH) and 150 mM KCl. For bulk crRNP synthesis, 10 μL of 160 μM crRNA was mixed with 10 μL of 160 uM tracrRNA, then incubated for 30 minutes at 37°C. The resulting crRNA:tracrRNA complexes were gently mixed with 20 μL of 40 μM *S. Pyogenes* Cas9 (UC-Berkeley Macrolab) then incubated at 37°C for 15 minutes. crRNPs were aliquoted into ten sets of 3.5 μL each and stored at −80°C. For pooled guide conditions, 5 independent crRNA targeting the same gene were mixed in equal volumes prior to tracrRNA addition. crRNAs were obtained from the Dharmacon predesigned Edit-R library or as custom sequences (see table of sequences below).

**Table:** 

Gene Target	Sequence	Catalog Number (Dharmacon)
NT #3	n/a	U-007503-20
NT #4	n/a	U-007504-20
CPSF6 #5	GGACCACATAGACATTTACG	CM-012334-05
CPSF6 #6	ATATATTGGAAATCTAACAT	Custom Sequence
CPSF5 #3	CAGGTTGATGGTGCGCTCCA	CM-012335-03
CPSF5 #12	CATGTGTTACTGCTGCAGCT	Custom Sequence
TRIM5α #6	AAGAAGTCCATGCTAGACAA	Custom Sequence
TRIM5α #7	GTTGATCATTGTGCACGCCA	Custom Sequence
CYPA #2	AGGTCCCAAAGACAGCAGGT	CM-004979-02
CYPA #3	GTACCCTTACCACTCAGTCT	CM-004979-03
CXCR4	GAAGCGTGATGACAAAGAGG	Custom Sequence
RPS2 #1–5 (Pool)	CGACCGACGCGTACCTTAAT, TACCTTGCAAGGGACAGTGT CACACTGTCCCTTGCAAGGT, AAGGCCGAGGATAAGGAGGT, CCTACCGAAGTTGCCCAGGG	CM-013690-01, CM-013690-02, CM-013690-03, CM-013690-04, CM-013690-05
IFNAR1 #1–5 (Pool)	CGCCACGGCGACGAGCACTA, AGTGTTATGTGGGCTTTGGA, GCTCGTCGCCGTGGCGCCAT, AGTGGATAATCCTGGATCAC, GATCTAATGTTAAAGACTGG	CM-020209-01, CM-020209-02, CM-020209-03, CM-020209-04, CM-020209-05

To generate polyclonal knock-out cells, 1×10^6^ cells per electroporation reaction were spun down at 400xg for 5 minutes and supernatant was removed by aspiration. Cells were resuspended in electroporation buffer consisting of 16.4 μL P3 Nucleofector solution with 3.6 μL supplement per reaction. 20 μL of cell suspension was mixed with 3.5 μL of each preformed crRNP and transferred to 96-well electroporation cuvettes for electroporation with the 4D Core Unit using pulse code EH-115 (Lonza). 100 μL warm cRPMI was added to each well following electroporation, and cuvettes were placed in cell culture incubator to recover at 37°C for 30 minutes. Cells were moved to 96-well flat bottom tissue culture plates prefilled with was added to each well following electroporation, and cuvettes were placed in cell culture incubator to recover at 37°C for 30 minutes. Cells were moved to 96-well flat bottom tissue culture plates prefilled with 100 μL complete RPMI, 2.5 μL T cell activation/expansion beads (Miltenyi Biotec, #130-091-441), and 0.2 μL of IL-2 (Miltenyi, #130097744). Cells were incubated at 37°C with 5% CO_2_.

### CRISPR-Cas9 Knock-in in HT1080 Cell Line

Endogenous monomeric NeonGreen (mNGreen) C-terminal tagged CPSF6 in HT1080 cell lines were generated by CRISPR-Cas9 knock-in. Briefly, antisense guide RNA (CACACATTTAACAGGGAACA) targeting sequences overlapping the stop codon of the CPSF6 gene locus was designed using the Benchling guide design online tool (Zang lab, MIT) and synthesized as lyophilized crRNA along with tracrRNA (IDT). The crRNA and tracrRNA were duplexed according to the manufacturer’s guidelines (IDT). 1 mM of the complexed sgRNA was mixed gently with 1μM of Alt-R *S. Pyogenes* Cas9 enzyme (IDT) in Opti-MEM (ThermoFisher, #11058021), incubated for 5 minutes at room temperature and stored in 4°C as RNP complex. A template of mNGreen gene, flanked by 800 base pairs homology arms complementary to the CPSF6 loci, was PCR amplified and cloned into Adeno-associated virus (AAV) using the NheI and XhoI restriction sites in the AAV backbone (Cell Biolabs) flanked by the AAV-ITRs to generate the pAAV-CPSF6-mNGreen construct. AAV particles containing CPSF6-mNGreen template were produced as previously described^47^. Briefly, plasmids encoding the recombinant AAV DJ capsid and replication genes (pAAV-DJ Rep-Cap, Cell Biolabs), helper (pAAV-Helper, Cell Biolabs), and knock-in template (pAAV-CPSF6-mNGreen) were transfected in 293AAV cells in a 1:1:1 ratio using 1 mg/mL polyethylenimine (PEI, Polysciences, #23966). 72 hours post transfection, the viral particles were harvested, filtered, aliquoted, and stored in −80. The sgRNA-Cas9 RNP complex was reverse transfected into 4×10^4^ HT1080 cells using RNAiMAX (ThermoFisher, #13778150) in a 96 well plate format and infected with 100 μL of AAV-containing CPSF6-mNGreen template. 48 hours post-transfection, cells were sub-cultivated in 48 well plate and sorted for single cell clones. Homozygous and heterozygous fluorescent knock-in CPSF6 cell clones were validated by PCR, sanger sequencing, western blotting and fluorescent widefield microscopy.

### Virus Stock Preparation

Replication competent was prepared using the original HIV-1 NL4–3 nef:IRES:GFP plasmid (NIH AIDS Reagent Program, #11349) in addition to the N74D and P90A capsid mutant plasmids described in the plasmid construction methods. For single round virus, a wild type or N74D capsid mutant HIV NL4–3 dEnv nef:IRES:GFP plasmid was pseudotyped with VSV-G. For virus production, low passage (<15) HEK293T cells were plated at a density of 5×10^6^ cells per 15 cm tissue culture dish 24 hours prior to transfection. 10 μg of plasmid DNA, 250 μL serum-free DMEM, and 30 μL of PolyJet (SignaGen Laboratories, # SL100688) were combined per plate of virus and transfected according to manufacturer protocol. 25 mL of viral supernatant was collected at 48 hours, replaced with fresh media, and a second set of supernatants were collected at 72 hours. Media was combined, filtered through 0.2 μM filters (Fisherbrand, #FB12566504), and incubated with 8.5% polyethylene glycol (Sigma Aldrich, #81260–5KG) and 0.3 M sodium chloride for 8 hours at 4°C. Viral precipitant was centrifuged at 3500 rpm for 20 minutes then resuspended in 250 μL DPBS resulting in 100x concentration. Virus aliquots were stored at −80°C prior to p24 viral titer quantification and use in infection assays.

### p24 ELISA for Viral Titer Quantification

Viral stocks were heat inactivated at 60°C for 30 minutes, then quantified using an HIV-1 Gag p24 Quantikine ELISA kit (R&D Systems, #DHP240B) according to manufacturer protocol. Samples were run in duplicate at dilutions of 1:500 and 1:2500. Viral stock concentrations were determined using a standard curve.

### HIV-1 Spreading Infection Assays

CRISPR-Cas9 knock-out primary CD4+ T cells were generated as described above. All experiments included knock-outs for HIV-1 co-receptor CXCR4, host factor cyclophilin A, and non-targeting controls alongside knock-out of other host factors of interest. At day 4 post-nucleofection, cells were split into replica plates of 1×10^5^ cells per well to generate samples for protein lysates for knock-out validation, viability assays, genomic DNA, HIV-1 infected protein lysates, and HIV-1 infection in triplicate per condition. Cells designated for protein lysates and viability assays were processed as described below. Genomic DNA was extracted using QuickExtract DNA Extraction Solution (Lucigen, #QE09050) according to manufacturer protocols and stored at −20°C. Plates designated for infection were infected in triplicate per condition with HIV-1 NL4–3 nef:IRES:GFP viral stocks diluted to a concentration of 0.2 ng p24 per 50 μL of complete RPMI with IL-2, and 50 μL of diluted virus was added to each well. Proper viral dose was determined by virus titration to optimize percent infection and cell viability (see **Extended Data Fig. 1c**). Infected cells were incubated in a cell culture incubator at 37°C with 5% CO_2_. 24 hours post infection, half of the cells received 50 μL of 25 μM Saquinavir (AIDS Reagent program, #NIH-ARP 4658) diluted in complete RPMI with IL-2. The remaining cells received an equal volume of dimethyl sulfoxide (DMSO) (Sigma Aldrich, #472301–100ML) diluted in media. At days 2 and 5 post infection, 75 μL of infected cell suspension was removed per well and fixed with 75 μL of 2% formaldehyde in DPBS for later analysis of infection by flow cytometry within 1 week post-fixation. Cells were fed with an additional 75 μL of complete RPMI supplemented with IL-2. Protein lysates were collected from the appropriate plate on day 5 post-infection.

For spreading infections conducted in the presence of cyclosporin A, cells were treated with cyclosporin A (Sigma-Aldrich, #SML1018) at a final concentration of 5 μM 4 hours prior to infection.

### Single Round Infection Assays

CRISPR-Cas9 knock-out primary CD4+ T cells were generated as described above using cells from 3 three independent blood donors. All experiments included knock-outs for HIV-1 co-receptor CXCR4, host factor cyclophilin A, and non-targeting controls alongside knock-out of other host factors of interest. At day 4 post-nucleofection, cells were split into replica plates of 1×10^5^ cells per well to generate samples for protein lysates for knock-out validation, viability assays, and HIV-1 infection in triplicate per condition. Cells designated for protein lysates and viability assays were processed as described below. Plates designated for infection were infected in triplicate per condition with viral stocks (HIV-1 NL4–3 nef:IRES:GFP or VSV-G-pseudotyped NL4–3 dEnv nef:IRES:GFP) at a concentration of 0.02, 0.05, 0.1, 0.2, 0.5, or 1 ng p24 per well suspended in 50 μL complete RPMI supplemented with IL-2. At day 2 post infection, 75 μL of cell suspension was fixed with an equal volume of 2% formaldehyde in DPBS for later analysis of infection by flow cytometry within 1 week post-fixation.

### Interferon Titration and Sensitivity Assays

Primary CD4+ T cells from three independent donors underwent CRISPR-Cas9 editing using guides targeting *CXCR4, CYPA, IFNAR1*, and *CPSF6* and replica plating as previously described. Additional replicates of 1×10^5^ cells per well in 96 well U-bottom plates were generated for the interferon titration assay. Plates for HIV-1 infection were treated with 100 U/mL universal type I interferon (PBL Assay Science, #11200–2), IFNα (PBL Assay Science, #11100–1), IFNβ (PeproTech, #300–02BC), or IFNγ (PeproTech, #300–02) or a media-only control 16 hours prior to infection. Following incubation with interferon, cells were infected with HIV-1 NL4–3 nef:IRES:GFP as described in spreading infection assay protocol. Infection timepoints were collected at days 2 and 5 post-infection, and day 5 HIV-1 infected protein lysates were collected as described below. To assess the impact of IFN treatment on ISG production, replica plated cells were treated with 10 U/mL of universal type I interferon, IFNα, IFNβ, IFNγ, or media-only controls. 16 hours post-interferon treatment, protein lysates were harvested according to protocol in immunoblotting methods for later analysis of interferon stimulated gene (ISG) expression.

### Receptor/Coreceptor and Activation Marker Surface Staining

*CXCR4, CCR5, CYPA, CPSF6*, and *TRIM5α* knockout cells and non-targeting control cells were generated using CRISPR-Cas9 editing as previously described using primary CD4+ T cells isolated from the blood of three independent human donors. 1×10^5^ cells per condition were used for cell surface marker staining for CXCR4 (APC, Miltenyi Biotec, #130-120-708 [clone REA649]), CD4 (PE, Miltenyi Biotec, #130-113-225 [clone REA623]), CD25 (APC, Miltenyi Biotec, #130-115-535 [clone REA945]), and CCR5 (APC, Miltenyi Biotec, #130-120-708 [clone REA245]) at day 5 post-editing. Antibodies were diluted in MACS buffer at a dilution of 1:50 and samples were stained for 15 minutes at 4°C in the dark. Samples were fixed in 1% formaldehyde in DPBS for analysis by flow cytometry within 1 week post-fixation. Flow analysis was performed using the gating strategy shown in **Supplementary Figure 1**.

### Viability assays

Cell viability was assessed at specified timepoints by Ghost Red amine dye stain and/or CellTiter-Glo assay. For Ghost Red stain, 1×10^5^ cells per condition were pelleted in 96-well U-bottom plates by centrifuging at 400xg for 5 minutes. Following a wash with DPBS, cells were resuspended in 100 μL of Ghost Red dye (Tonbo Biosciences, #13–0871-T100) diluted 1:1000 in DPBS. Cells were incubated in the dark at 4°C for 30 minutes, then spun down and supernatant was aspirated. Cells were washed with MACS buffer (DPBS (Corning, #21–031-CV) with 0.5% BSA (Fisher Scientific, BP9706100) and 2mM EDTA (Corning, #46–034-Cl)), spun down, and resuspended in 150 μL 1% formaldehyde in PBS for later analysis by flow cytometry.

For CellTiter-Glo Assay, cells in 96 well U-bottom plates were resuspended in 100 μL media and moved to an opaque, white 96 well plate (Corning, #3912). Cells were incubated at room temperature for 30 minutes to equilibrate to room temperature. Cell suspension was mixed with 100 μL of room temperature CellTiter-Glo reagent (Promega, #G7570) and placed on an orbital shaker for 2 minutes to complete cell lysis. Plate was incubated 10 additional minutes at room temperature to stabilize luminescence signal before reading luminescence on an Omega plate reader (BMG LabTech), with a consistent gain setting used across readings.

### Immunoblotting

Primary CD4+ T cell protein lysates were generated by washing 1×10^5^ cells per condition in DPBS, removing supernatant, and resuspending cells in 50 μL 2.5x Laemmli Sample Buffer (1.9 mL 0.5 M Tris-HCl pH 6.8 (Fisher Bioreagents, #BP153–1), 6 mL 50% glycerol (Fisher Bioreagents, #BP229–1), 3 mL 10% SDS (Corning, #46–040-CI), 250 μL β-mercaptoethanol (Fisher Chemical #O3666I-100), 50 μL 1% bromophenol blue (Fisher Bioreagents, #BP115–25), 18.8 mL DPBS (Corning, #21–031-CV)). Lysates were heated at 98°C for 20 minutes, then stored at −20°C prior to use for immunoblotting. Lysates were run on 4–20% Criterion Tris-HCl 18 or 26 well gels (Bio-Rad, #3450033 and # 3450034, respectively) at 90V for 30 minutes followed by 150V for 70 minutes. Proteins were transferred to PVDF membranes (Bio-Rad, #1620177) for 2 hours at 90V. Membranes were blocked in 4% milk in PBS (Sigma-Aldrich, #P5493–4L) with 0.1% Tween-20 (Fisher Bioreagents, #BP337–500) or 5% BSA in PBS with 0.1% Tween-20 according to antibody manufacturer recommendations for 1 hour, then incubated with primary antibody overnight at 4°C with shaking. Primary antibodies used in this study are as follows: CPSF6 (Rabbit, 1:3000 in 5% BSA, Novus, #NBP1–85676), CPSF5 (Rabbit, 1:5000 in 4% milk, Proteintech, #10322–1-AP), CYPA (Rabbit, 1:12000 in 4% milk, Cell Signaling, #2175), IFNAR1 (Rabbit, 1:1000 in 4% milk, abcam, #ab124764 [clone EPR6244]), MX1 (Rabbit, 1:1000 in 5% BSA, Cell Signaling, #37849S [clone D3W7I]), TRIM5α (1:1000 in 4% milk, Cell Signaling, #14326 [clone D6Z8L]), and β-actin (Mouse, 1:10000 in 4% milk, Cell Signaling, #3700S [clone 8H10D10]). Membranes were washed with PBS with 0.1% Tween-20, then incubated with either anti-mouse (Peroxidase AffiniPure^™^ Goat Anti-Mouse IgG (H+L), Jackson ImmunoResearch, #115-035-003) or anti-rabbit (Peroxidase AffiniPure^™^ Goat Anti-Rabbit IgG (H+L), Jackson ImmunoResearch, #111-035-003) HRP-conjugated secondary antibodies at 1:10000 in 4% milk in PBS with 0.1% Tween-20 for 1 hour, washed with PBS with 0.1% Tween-20, then detected using Immobilon Western Chemiluminescent HRP Substrate (EMD Millipore, #WBKLS0500) and imaged using an iBright imaging system (Thermo Fisher). Blots were incubated in antibody stripping solution (EMD Millipore, #2502) prior to re-probing. Blots were quantified by measuring the rolling ball-corrected density of each analyzed band using iBright Image Analysis Software (version 5.2.1.0). Density values for the target protein bands (CPSF6 or MX1) were divided by density values for β-actin loading control bands, and the resulting ratio was normalized to the non-targeting ratio for each donor.

### Flow Cytometry

Flow cytometry analysis was performed using an Attune NxT acoustic focusing cytometer and CytKick Max Autosampler attachment (Thermo Fisher Scientific). Samples were suspended in 150 μL DPBS with 1% formaldehyde and were subjected to one 150 μL mixing cycle. All events in 50 μL of sample were recorded. FCS3.0 files were exported using Attune NxT Software (version 5.3.0) and analyzed using a FlowJo (version 10.10) template shown in **Supplementary Figure 1** for HIV-1 infection (BL1:GFP), Ghost Red Stain (RL-2), or cell surface receptor stain (RL-1: CXCR4-APC, CCR5-APC, CD25-APC, BL-2: CD4-PE).

### HIV-1 Infection and Live Cell Sorting for RNA-seq

21×10^6^ stimulated primary CD4+ T cells from donors X-Z were infected with wild-type or N74D capsid mutant HIV-1 NL4–3 nef:IRES:GFP alongside uninfected controls. Briefly, cells were resuspended in complete RPMI supplemented with IL-2, concentrated HIV-1 stocks for the appropriate virus were added to the cell suspension at a dose of 210 ng per condition, and the final volume of media was brought to 42 mL. Cells were then distributed into 96 well U-bottom plates, with each well containing 200 μL media, 1×10^5^ cells, and 1 ng p24. 1×10^6^ cells per donor were distributed into 96 well U-bottom plates at a concentration of 1×10^5^ cells per well in 200 μL complete RPMI with IL-2 to generate uninfected control cells. All cells were incubated in a cell culture incubator at 37°C with 5% CO_2_. On day 2 post-infection, infected cells were pooled per donor and virus strain. 1×10^6^ uninfected cells per donor immediately underwent RNA extraction according to protocol below to generate the uninfected RNA samples. The remaining infected cells (~20 million) were spun down at 400xg for 5 minutes, resuspended in 1 mL MACS buffer, and transferred to MACSQuant Tyto Cartridges (Miltenyi Biotec, #130-104-791). Cells in cartridges were immediately sorted using a MACSQuant Tyto sorter (Miltenyi Biotec) with MACSQuantify Tyto Software (version 2.0). Cells were sorted by gating for infection (GFP+) status as described in **Supplementary Figure 1**. The cartridge mixer was set at a speed of 800 rpm, and the cell sorting pressure was set to 150 hPa. Following sorting, cells were resuspended in 200 μL MACS buffer and retrieved from the sorted cartridge chamber. Samples were spun at 400xg for 5 minutes, supernatant was aspirated, and RNA was extracted according to the protocol below.

### RNA Extraction

RNA was extracted from donors E-G (Figure 3) at day 5 post nucleofection and donors X-Z (Figure 4) at day 2 post-infection or day 2 post-nucleofection depending on the sample. RNA was extracted from cell pellets using RNeasy Mini Kit (QIAGEN, #74104) according to manufacturer protocol. Samples were treated with DNAse I according to manufacturer protocol (QIAGEN, # 79254) and eluted in RNase-free water. RNA concentration was determined by NanoDrop (Thermo Scientific), and samples were stored at −80°C prior to analysis.

### HT1080-CPSF6-mNGreen HIV-1 Infection and RNA Extraction

1×10^5^ HT1080-CPSF6-mNGreen cells per condition were seeded in 12-well plate and adhered to plates overnight prior to infection. In parallel, 3×10^4^ HT1080-CPSF6-mNGreen cells were seeded in 8-well chamber slides (Ibidi, #80806) coated with 0.1 mg/ml poly-D lysine hydrobromide (Advanced Biomatrix, #5049). These cells were infected with VSV-G pseudotyped wild type or N74D capsid mutant HIV-1-NL4–3 at an MOI of 10 or mock-infected by spinoculation at 16°C for 1 h at 1200xg. Cell cultures were washed once with prewarmed DMEM and returned to a tissue culture incubator at 37°C with 5% CO_2_ for 6 hours. RNA from wild type, N74D capsid mutant HIV-1-NL4–3, and mock infected cells in the 12-well plate was then extracted using a NucleoSpin RNA Mini kit (Machery-Nagel, # 740955.50) according to manufacturer’s protocol. RNA concentration was determined by NanoDrop (Thermo Scientific), and samples were stored at −80°C prior to analysis. In parallel, HIV-1-NL4–3 wild type, N74D and mock infected cells HT1080-CPSF6-mNGreen infected cells on the 8-well chamber slides were washed with 1x DPBS, fixed with 4% formaldehyde (Sigma, Cat# P6148–1KG) and imaged by widefield deconvolution fluorescent microscopy (GE, DeltaVision OMX SR Imaging System).

### RNA-Sequencing

RNA samples from donors E-G (Fig. 2) were submitted to the NUSeq core for next-generation sequencing. Libraries were prepared using TruSeq Total RNA-Seq Library Prep (Illumina) and sequenced using an Illumina NovaSeq 6000 with 150 bp paired-end reads. RNA samples from donors X-Z (Fig. 5) were submitted to Novogene for next-generation sequencing. Libraries were prepared using NEBNext Ultra II kit with poly(A) selection according to manufacturer protocols (New England Biolabs). Samples were sequenced using an Illumina NovaSeq 6000 with 150 bp paired-end reads. RNA samples from HT1080 cells (Fig. 5) were submitted to Novogene for next-generation sequencing. Libraries were prepared using NEBNext Ultra II kit with poly(A) selection according to manufacturer protocols (New England Biolabs). Samples were sequenced using a NovaSeq X Plus with 150 bp paired-end reads.

### RNA-Sequencing Data Processing

The RNA-seq data processing pipeline was adapted from published protocols^48^. Briefly, we first trimmed the paired-end reads in FASTQ files using Trimmomatic (version 0.39)^49^, targeting known adapters (TruSeq3-SE.fa:2:30:10), and also trimming the first and last 3 base pairs if their quality fell below set thresholds. Additionally, we employed a 4-base sliding window technique, trimming reads when the average window quality dropped below 15. Reads shorter than 36 base pairs were excluded. Next, the trimmed reads were aligned to the human reference genome GRCh38 (https://www.ncbi.nlm.nih.gov/datasets/genome/GCF_000001405.26/) using HISAT2^50^ (version 2.1.0) and converted into BAM format using samtools (version 1.6)^51^. The alignment data was then processed by StringTie (version 2.1.3)^52^ to estimate gene expression levels. The StringTie merge function merged samples’ gene structures to create a unified set of transcripts across all samples. Finally, read counts were obtained using prepDE.py (https://ccb.jhu.edu/software/stringtie/index.shtml?t=manual) to prepare the data for DESeq2.

### Differential Gene Expression Analysis

The raw read counts were processed in R (version 4.2.3). Initially, transcript names were converted to gene symbols, eliminating duplicate symbols and dropping transcripts that did not map to known gene symbols. The resulting reads were used to construct a DESeqDataSetFromMatrix object using DESeq2 (version 1.38.3)^53^ for investigating differences across conditions, accounting for donor variability. Genes with a minimum count of 10 in at least 3 samples were retained for analysis. To visualize treatment effects, counts were transformed using the variance stabilizing transformation (VST) function from DESeq2 and subjected to principal components analysis (PCA), visualizing the first two principal components^54^.

Differential gene expression was assessed using the DESeq function from DESeq2, which estimates size factors and dispersions, fitting a negative binomial generalized linear model. P-values were adjusted using the Benjamini-Hochberg method^55^, considering adjusted p-values below 0.05 as significant. Subsequently, complete cases were filtered, and the lists of differentially expressed genes were input into Metascape (version 3.5.20240101) for identifying pathways associated with up- or downregulated gene sets^56^. The log fold change for genes identified by Metascape that were associated with the interferon gamma signaling pathway were visualized for all 3 donors (individually and collectively) in a heatmap. The top 2000 significant genes with highest log fold changes were also Z-score transformed and clustered using hierarchical clustering that was visualized in a heatmap^57^.

### Alternative Polyadenylation Analysis

APA analysis was conducted by analyzing RNA-seq data using the REPAC pipeline (version 0.99.0) to determine changes in 3’UTR length in treated *(CPSF6* knock-out or infected) versus control (non-targeting or uninfected) conditions^37^. 3’ UTRs were designated shortened for Compositional Fold Change (cFC) < 0.25 and −log_10_(adjusted p-value) < 0.05 and lengthened for Compositional Fold Change (cFC) > 0.25 and −log_10_(adjusted p-value) < 0.05. Lists of genes with significant changes in 3’ UTR length were input into Metascape (version 3.5.20240101) for identifying pathways associated with genes sets with shortened or lengthened 3’ UTRs, and the list of all genes with tested APA sites was provided as a background gene set^56^.

Additional validation of 3’UTR length changes were carried out using Dapars (Dynamic analysis of Alternative PolyAdenylation from RNA-seq) (version 0.9.1)^58,59^. Distal polyadenylation sites (PAS) for each gene were extracted from the GRCh38/hg38 refseq gene model downloaded from UCSC (https://genome.ucsc.edu/). A proximal PAS for each gene was determined by identifying the optimal fitting point of a linear regression model using alignment wiggle files for each sample created using BedTools (version 2.30.0)^60^. Differential PAS usage was then quantified as a change in Percentage of Distal PAS Usage Index (ΔPDUI) for each gene in treated *(CPSF6* knock-out or infected) versus control (non-targeting or uninfected) conditions, using default parameters. Genes were considered significantly shortened or lengthened if they had a false discovery rate (FDR) of <0.05.

RNA-Seq read tracks were visualized using Integrated Genomics Viewer (version 2.17.4) to show changes in 3’ UTR read coverage for genes of interest *(TGFBR1, IL2RA/CD25, CXCR4)* using RNA-Seq data from Donor E. Track heights were kept consistent across samples. Violin plots depicting changes in 3’ UTR length among gene sets of interest (Cytokine Signaling in Immune system (R-HSA-1280215), Interferon signaling (R-HSA-913531), and T cell activation (GO:0042110)) were generated by extracting gene names associated with the gene sets using biomaRt (version 2.58.2) and annotating the output of the REPAC and ΔPDUI analyses to indicate which of the tested genes were a member of the gene sets.

### Statistics

All statistical analysis was performed using GraphPad Prism version 10.2.1 (395) for Windows, GraphPad Software, Boston, Massachusetts USA, www.graphpad.com and R (version 4.2.3) stats package (version 3.6.2). All statistical analysis performed in this manuscript is available in **Supplementary Table 1**.

## Data Availability

Deposition of the raw RNA-Seq data from experiments performed using CD4+ T cells isolated from the blood of human donors into public databases is restricted. All processed RNA-sequencing data from experiments performed using human primary CD4+ T cells is provided as a spreadsheet (**Supplementary Table 2**). Sequencing files for RNA-Seq experiments with HT1080 cells are deposited to NCBI as Bioprojects. All other data are available from the corresponding author upon request.
